# Molecular Identification of Human Papillomavirus DNA in Thyroid Neoplasms: Association or Serendipity?

**DOI:** 10.7759/cureus.14578

**Published:** 2021-04-20

**Authors:** María Ivette Muciño-Hernández, Héctor Montoya-Fuentes, Miguel Ricardo Ochoa-Plascencia, Gonzalo Vázquez-Camacho, Elías Adrián Morales-Jeanhs, Alfonso Enrique Bencomo-Álvarez, Jonathan Matias Chejfec-Ciociano, Clotilde Fuentes-Orozco, Francisco José Barbosa-Camacho, Alejandro González-Ojeda

**Affiliations:** 1 Medical Sciences Postgraduate Program, University of Colima, Colima, MEX; 2 Molecular Microbiology Laboratory, Biomedical Research Center 01, Western National Medical Center, Mexican Institute of Social Security, Guadalajara, MEX; 3 Biomedical Sciences Postgraduate Program, Health Sciences University Center, University of Guadalajara, Guadalajara, MEX; 4 Basic Science, School of Medicine, Instituto Tecnológico de Estudios Superiores de Monterrey, Monterrey, MEX; 5 Biomedical Research Unit 02, Specialties Hospital - Western National Medical Center, Mexican Institute of Social Security, Guadalajara, MEX

**Keywords:** human papillomavirus, polymerase chain reaction, thyroid neoplasms

## Abstract

Background: Human papillomavirus (HPV) is recognized as the most important cofactor in the etiology of cancers of the cervix, esophagus, larynx, and nasopharynx. Experimental evidence suggests that HPV could have an oncogenic influence on thyroid follicular cells; however, to the best of our knowledge, there is no record of its role in human thyroid gland neoplasms.

Objective: The purpose of this study is to describe the frequency and the types of HPV present in neoplastic thyroid tissue by polymerase chain reaction-restriction fragment length polymorphism (PCR-RFLP).

Methods: Over 157 samples were analyzed of paraffin-embedded tissue from malignant and benign thyroid tumors. All the paraffin blocks were selected consecutively from the Pathology Tissue Bank archive of the Western Medical Center. The molecular detection and typing were performed at the Molecular Microbiology Laboratory of the Biomedical Research Center, Mexican Institute of Social Security.

Results: The frequency of HPV findings was 2.5% (four cases). HPV-6 was found in two cases of thyroid hyperplasia (2.5%), and HPV-33 in two cases of papillary cancer (4.6%).

Conclusion: The presence of HPV is not frequent in thyroid neoplasms, at least in the studied population. Due to the low prevalence of this virus in our sample, it is not possible to reach conclusions. Further research is needed.

## Introduction

The discovery of the virus’s role in the carcinogenic process marked a fundamental point in understanding these pathologies. We currently know that 12% of neoplasms worldwide are caused by a previous viral infection out of which the human papillomavirus (HPV) is responsible for 30% [1]. This event has been well documented across various neoplasms, including those of the cervix, larynx, prostate, bladder, breast, esophagus, and colon [2]. Thyroid tumors are found frequently among the general population. The incidence of benign thyroid tumors and thyroid cancer has increased in recent years. In 2018, the incidence of thyroid cancer in Mexico was 12,200 cases [3]. Despite the high frequency of thyroid tumors, current literature regarding HPV frequency in neoplastic thyroid tissue and the relationship with thyroid tumors appears scant.

HPV and its role in head and neck cancer

HPV is an epitheliotropic, small, double-stranded DNA virus, with more than 100 typified subtypes, 15 of them highly oncogenic. In the last 30 years, an increase in the prevalence of HPV infections has been observed, along with a close relationship with several oncogenic processes. An association between cancer and the presence of HPV has been recognized in young people (<45 years old) without a history of alcoholism or smoking. HPV types 16 and 18 are identified in premalignant lesions (leukoplasia). However, their transmission and cellular transformation mechanisms are not yet fully understood [4].

Head and neck squamous cell carcinoma represent about 6% of all types of cancer [1]. Two meta-analyzes in China evaluated the presence of HPV-16 and HPV-18 in different neoplasms of the head and neck. HPV 18 was found in 6% of the 1881 samples with an incidence of up to 31.2% in laryngeal cancer. On the other hand, HPV-16 had an incidence of 24.7% in the 2896 samples, with 28.5% in oropharyngeal cancer [5,6]. Gallegos-Hernández et al. identified HPV-16 in 42% of Mexican patients with severe head and neck malignancies (n = 118). HPV could be considered as a cofactor for oral, laryngeal, and esophageal carcinogenesis because of its presence (and the previously described effects) on the cell division cycle.

Thyroid tumors

Thyroid tumors have their origin in the epithelium, arising from the follicular or parafollicular cells. They are the most common endocrine malignant tumor, representing 1.1% of all malignancies [3]. By contrast, the frequency of benign tumors, such as adenoma or hyperplasia, is increasing. In 2006, a Mexican study of 300 cadavers without known thyroid pathology found a prevalence of thyroid nodules of 41%, of which 4% had a histopathological diagnosis of malignancy [7].

Various studies have investigated the role of several viruses in the thyroid cancer process. Among them, a meta-analysis that includes 23 studies concluded that the presence of some viruses increases the risk of development of thyroid cancer, some of the viruses with greater evidence are Epstein-Barr viruses (EBV), human parvovirus (B19), BK virus (BKV), human herpes simplex virus (HSV), and hepatitis C virus [8]. Among the types of thyroid tumors, the papillary is the one most associated with viral infections, being the most common, representing 80% of all cases [8]. The relationship between the Epstein-Barr virus and thyroid cancer is well-established. A study carried out in 2019 found the presence of the virus by polymerase chain reaction (PCR) in 71.9% of the 57 patients, together with higher IL-6 and NF-KB values than the control group [9]. Several studies have established the role of the virus in the activation of inflammatory genes, disruption of the cellular signaling, secretion of cytokines, and the creation of an environment that avoids anoikis, a process of cell death related to the extracellular matrix. These characteristics allow the evolution and uncontrolled growth of the tumor which might also define the state of cancer [10,11].

Due to the strong relationship between this virus and the oncological processes reported in multiple organs, the aim of this study was to describe the frequency of onset of thyroid tumors, reveal the types of HPV present in malignant and benign neoplastic thyroid tissue by polymerase chain reaction-restriction fragment length polymorphism (PCR-RFLP), and to unveil the presence of HPV in thyroid tissue. This article was previously presented as an abstract at the 2011 Official Program Abstracts, Otolaryngology-Head and Neck Surgery journal.

## Materials and methods

Sample collection

We included 157 thyroid tumor tissues (benign: adenoma and hyperplasia; malignant: papillary, follicular, and anaplastic cancer) from paraffin-embedded tissue blocks in this study. The block selection was done consecutively from the archive of the Pathology Tissue Bank of the Western National Medical Center, Mexican Institute of Social Security between January 2003 and December 2007.

In all cases, the paraffin blocks and slides were reviewed by a pathologist to reaffirm the diagnosis. The most representative blocks according to the tumor type were chosen. From the clinical file, epidemiological data such as age, sex, and history of alcoholism/smoking were obtained.

The DNA of the samples that fulfilled the inclusion criteria, including a histopathological diagnosis of papillary cancer, or follicular or anaplastic thyroid cancer in any cancer stage, was extracted and processed for PCR in the Molecular Microbiology Laboratory of the Western Biomedical Research Center-Mexican Social Security Institute. Samples with more than one diagnosis on file, with a family record of cancer, or any radiation exposure were excluded.

Sample management and DNA extraction

DNA Extraction

For DNA extraction, the Wright and Manos protocol was used [12]. Tissue fragments were obtained with a scalpel blade and introduced in 2 mL Eppendorf tubes. The blocks were exposed to 1 mL of octane (Acros Organics, Fisher Scientific Division, Pittsburgh, PA, USA) at room temperature for 30 minutes to remove paraffin wax. The tubes were then centrifuged for five minutes at 10,000 rpm. The supernatant was discarded and the octane addition was repeated. Subsequently, 500 ìL of 100% ethanol was added to each tube, the contents mixed gently by inversion, and then centrifuged for five minutes at 10,000 rpm. This step was repeated. The alcohol supernatant was discarded, and 60 ìL of acetone was added; the tubes were kept open for air drying at 37 °C until all the solvents had evaporated.

Subsequently, 100 ìL of lysis buffer (50 mM Tris base, pH 8.5, 1 mM EDTA, 0.5% Tween 20) with 200 ìg/ml of Proteinase K (Invitrogen, Carlsbad, CA, USA) were added and mixed gently with the tube contents and incubated at 37 °C overnight. The tubes were then fast centrifuged for 10 s, the aqueous phase removed and collected in 0.2 mL Eppendorf tubes, and the Proteinase K was heat-inactivated in a thermal cycler program. The extracts were kept frozen at −20 °C.

Polymerase Chain Reaction

The extracted DNAs (previously measured in terms of quantity and quality by spectrophotometry) were adjusted to a concentration of 100 ng/ìL and amplified with CpI or CpIIG consensus primers (CpI: 5′-TTA TCA WAT GCC CAY TGT ACC AT-3′, CpIIG: 5′-ATG TTA ATW SAG CCW CCA AAA TT-3′), to amplify a 188 bp fragment from the E1 ORF of several HPVs. As an internal control, specific primers for the amylin constitutive gene were used (FE3: 5′-TCA CAT TTG TTC CAT GTT AC-3′, Clg1: 5′- TCT AAA GGG GCA AGT AAT TCA GT-3′) [13].

The PCR was conducted as previously described by our laboratory in a 32-cycle program (Table 1). After the PCR, the amplified products were kept at 4 °C. The PCR products were separated and confirmed by polyacrylamide gel electrophoresis (6%, 29:1) in 1× TBE buffer. Gels were stained using a silver nitrate protocol previously described by Sanguinetti et al. [14].

**Table 1:** Polymerase chain reaction conditions for human papillomavirus amplification. dATP: deoxyadenosine triphosphate, dCTP: deoxycytidine triphosphate, dGTP: deoxyguanosine triphosphate, dTTP: deoxythymidine triphosphate.

Reagent	Initial concentration	Final concentration	Volume/20 ìL
PCR buffer	10×	1×	2 ìL
MgCl_2_	50 M	1.5 M	0.6 ìL
dATP	10 M	0.2 ìL	0.4 ìL
dCTP	10 M	0.2 ìL	0.4 ìL
dGTP	10 M	0.2 ìL	0.4 ìL
dTTP	10 M	0.2 M	0.4 ìL
CpI primer	10 p/m/ìL	1 p/m/ìL	2 ìL
CpIIG primer	10 p/ml/ìL	1 p/ml/ìL	2 ìL
Taq-pol	5 U/ìL	5 U/ìL	0.1 ìL

HPV Typing

The HPV-positive amplified products were digested with the restriction enzyme RsaI, which recognizes and cleaves the sequence 5′-GT/AC-3′ in several sites of the consensus 188 bp fragment. The obtained patterns are characteristic for the most part of the detected HPV types [15], which allow identifying more than two HPV types in a single sample. Among those viruses, the system can identify HPV-6, 11, 16, 18, 31, 33, 39, 51, and 58, and viruses related to recurrent respiratory papillomatosis, cervical uterine cancer, and other tumors [13,15].

The 188 bp fragments of the positive samples were recovered from the gel, using a sterilized scalpel blade for each sample, and placed in a 0.5 Eppendorf tube with 50 ìL of recovery buffer (10 mM Tris-HCl pH 9.0, 5 mM KCl, 1.5 mM MgCl_2_, 0.1% Triton X-100) and incubated for 20 minutes at 95 °C. After reamplification with the same primers (CpI and CpIIG), 10 ìL of the second amplified product with the addition of restriction enzyme RsaI were incubated, according to the manufacturer’s protocol (Invitrogen, Carlsbad, CA, USA). To observe the restriction fragment pattern for each sample, high-density polyacrylamide gel electrophoresis (12%, 19:1) was performed, using 10 bp ladder and pBR322 HaeIII-digested plasmid as molecular weight markers, placed in the ends and in the middle of the gel to ensure an accurate estimation of the restriction fragments for the typing [13].

Quality control of each procedure was conducted according to well-known standard protocols, such as the inclusion of DNA blanks and positive samples for each analysis and the use of a reporter gene (amylin). We used 10% sodium hypochlorite and 1% SDS solutions to decontaminate work areas before conducting the procedures.

Statistical analyses

The results were analyzed using IBM SPSS Statistics for Windows (version 20, IBM Corp, Armonk, NY, USA). The descriptive analyses of the pathological and clinical variables are shown as means and standard deviations.

## Results

We analyzed 157 thyroid tumor samples using PCR-RFLPs. Table 2 shows the general data for the patients such as age and sex, according to the tumor type.

**Table 2:** Demographic characteristics and type of thyroid neoplasms.

Type of tumor		Sex (%)			Age (years ± SD)		
		Male	Female	Total	Male	Female	Mean ± SD
Benign	Adenoma	3 (1.9%)	20 (12.7%)	23 (14.6%)	45.3 ± 11.7	56.5 ± 7.4	52.7 ± 9.9
Benign	Hyperplasia	12 (7.6%)	67 (42.7%)	79 (50.3%)	64 ± 9.8	49.7 ± 14.9	51.8 ± 15.2
Malignant	Papillary cancer	10 (6.4%)	33 (21%)	43 (27.4%)	49.8 ± 10.2	49.6 ± 12.7	49.7 ± 12.1
Malignant	Follicular cancer	2 (1.3%)	6 (3.8%)	8 (5.1%)	30 ± 2.8	26.6 ± 2.9	28 ± 3.1
Malignant	Anaplastic cancer	2 (1.3%)	2 (1.3%)	4 (2.6%)	45 ± 36.7	65.5 ± 0.7	55.2 ± 24.3
Total		29 (18.5%)	128 (81.5%)	157 (100%)	56.7 ± 15.5	49.9 ± 14.3	51.1 ± 14.6

Nineteen patients (12.1%) had a history of smoking. In all those cases, the tumor lesion (malignant or benign) was completely resected during the surgical intervention. Seven of the patients with papillary cancer, each older than 45 years, showed metastasis to cervical lymph nodes (from 2 to 5), which were totally resected (stage III). Two patients with papillary cancer and three with hyperplasia were declared deceased because of nonthyroidal pathology-related causes, between one and three years after surgery. The four patients with anaplastic cancer died within one year after their diagnosis.

Table 3 shows the HPV-type cases according to the tumor type. Four HPV-positive samples were found (HPV-33 in two papillary cancer tissues and HPV-6 in two hyperplasias). All four cases were in women older than 45 years and with nonprogressive disease (papillary cancer). In those patients, the history of cervical dysplasia or cancer because of HPV (through the routine preventive program on adult Mexican women) was negative. The cases HPV positive (both benign and malignant) did not show a different clinical behavior nor characteristic morphologic changes from those HPV negative.

**Table 3:** Tumor types, human papillomavirus positivity, and viral subtype.

Tumor type		Sample (%)	Positivity (%)	Subtype
Benign	Adenoma	23 (14.6%)	0	
	Hyperplasia	79 (50.3%)	2 (2.5%)	6
Malignant	Papillary cancer	43 (27.4%)	2 (4.6%)	33
	Follicular cancer	8 (5.1%)	0	
	Anaplastic cancer	4 (2.6%)	0	
Total		157 (100%)	4 (2.5%)	

**Figure 1:**
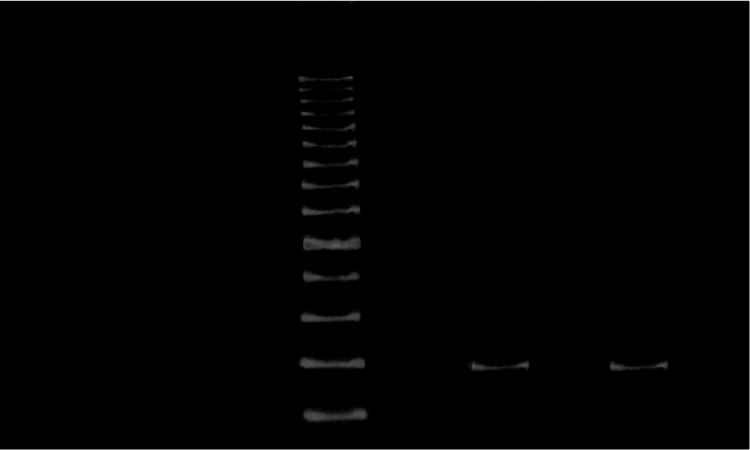
Polyacrylamide gel electrophoresis of polymerase chain reaction products. Lane 7: HPV positive sample (188 bp fragment); lanes 1, 2, 3, 4, 6: HPV-negative samples; lane 5 corresponds to a 100 bp ladder as a molecular weight marker; lane 8 shows a negative control; line 9 shows an HPV-16 positive control.

Figure 1 shows an HPV-positive sample electrophoresis corresponding to papillary cancer.

## Discussion

The average age of the patients sampled was 51.1 ± 14.6 years, which is consistent with previous findings that women are most affected by thyroid tumors and are commonly between their fourth and sixth decade of life [3]. All of those data are in agreement with the reports of the local Health Ministry in 2006 [16] for those types of tumors.

A history of smoking was found in 12.1% of the patients. The estimated smoking prevalence in the local population is considerably higher in men (40%) than in women (13.4%). There is no conclusive evidence that smoking is a determinant risk factor for thyroid tumors [17].

Patients who showed papillary cancer metastasis to lymph nodes could be considered in stage III of the disease because of their age (>45 years old), as stated by the American Academy of Cancer [3]. The 10-year survival prognosis is >90%. By contrast, patients showing anaplastic lesions have a survival prognosis at a diagnosis of just six months [3]. This type of cancer appears around the sixth to seventh decade of life; however, one of the samples of the study was from a 19-year-old male patient, with the same course of evolution.

The present work redresses the relative absence of previous studies of the possible role of HPV in thyroid tumors at the time of its initiation. We found four HPV-positive cases (2.5%). Two cases with hyperplasia were HPV-6 positive, and two papillary cancer cases were HPV-33 positive, both types previously described in oropharynx cancer, with frequencies ranging between 10% and 60% [15,18,19].

Subtypes HPV-6 and -11 have mainly been associated with premalignant lesions and condyloma, and have been reported in nose, mouth, larynx, and conjunctiva mucosa [4]. In the present study, HPV-6 was found in two cases of thyroid hyperplasia, a benign tumor characterized by the diffuse growth of follicular cells and multiple etiologies.

The two cases of papillary cancer were HPV-33 positive, which is considered as a high-risk type of HPV, mainly associated with oropharynx and cervical cancer [19]. Both cases of papillary cancer were found in women >45 years old and without a history of anogenital HPV infection or dysplasia.

Every HPV type shows a specific preference for cutaneous or mucosa epithelia. In Mexico, the most prevalent HPV types are 16 and 18, mainly in the cervical pathology and in extra cervical sites, such as the pharynx, larynx, esophagus, breast, and retinoblastoma [12,13,15]. We found no positive samples in this study with such types.

The preference of the HPV for mucosa and epithelia and their oncogenic role is well known. HPV targets the germinal cells in the lower third of the epithelium, achieved through microbursts, lacerations, or other traumatic events. During the initial infection, given that the virus has no capacity for self-replication, it relies on the help of oncoproteins and the inhibition of regulatory genes to induce the replication phase to the host cell and gain access to its DNA polymerase. The proliferation activity together with the oncoproteins give rise to cellular stress that can end in chromosomal instability and the beginning of a cancerous process. The high prevalence of this virus in neoplastic lesions is due to the ability not only to provide cellular stress, but also metabolic changes that help promote, progress, and metastasize cancer [1]. Certain HPV types may appear in malignant lesions as a result of coinfection and may not be the etiological agent of the tumor [20].

HPV associated with a high risk of cancer has been found in 99.7% of cervical tumors. Uterine cervix-affecting HPV types are usually associated with other genital tumors, affecting both genders (penis, vulva, vagina, and anus) and some neoplasms of the oral cavity, oropharynx, and skin [21]. In the oral cavity, HPV can be found in the tongue, palate, gums, and lips. However, malignization of those oral lesions is less frequent [22].

Recently, the search for HPV in lesions affecting extragenital sites, such as the breast and prostate, has revealed possible roles of HPV in such tumors [23-25]. The frequency of HPV in head and neck squamous cancers is variable. The results of the meta-analysis suggest that this variability could be caused by ethnogeographic differences among the studied populations, analytical sensitivity of the detection method, specimen preservation protocols, and site of the lesion. Moreover, the reported frequency is inversely proportional to the sample size [26].

The mechanism of HPV infection in nonepithelial tissue or mucosa (thyroid) remains unclear [21]. The spread of the virus via the bloodstream through monocytes could explain the nonsexual HPV dissemination, as Bodaghi et al. suggest [27]. There is evidence for HPV-positive peripheral blood in patients with an advanced degree of cervical and head and neck cancers, especially in metastasis [4].

Stamatiou et al. recently searched for polyomavirus (VP1), EBV, and HPV in 30 frozen specimens of thyroid nodules and normal thyroid tissue. They found VP1 in 60% (18 out of 30) of thyroid cancers or multinodular hyperplastic lesions, compared with 43.3% (13 out of 30) of adjacent normal thyroid tissue specimens. In no case did they find either EBV EBER1 sequences or HPV DNA [28].

The effect of HPV on thyroid neoplasms has been reported only for studies using transgenic mice, in which follicular thyroid cells were transfected with HPV-E7 ORF and HPV-16. [11]. The a posteriori observations in the first month were proliferative changes in cells and neoplastic changes within a year of the study, which are features compatible with papillary cancer. These observations show the plausibility of a role for HPV in the pathogenesis of thyroid tumors [11]. Notably, we found no positive cases for these types of HPV.

The oncogenic properties of HPV-E7 protein are well known. Interaction of HPV-E7 with RB1, and its subsequent inactivation, results in continuous thyroid cell growth, which can alter their differentiation, function, and regulation [29]. Additional studies are required to evaluate the oncogenic role of other HPVs in thyroid cancer pathogenesis.

It appears feasible that HPV infection participates in the pathogenesis of thyroid tumors, which are unrelated to those of epithelial or mucosal origin. The possibility of low-quality DNA because of the paraffin-embedded tissue source being responsible for the low frequency of HPV findings is not unreasonable. The quality and quantity of the extracted DNA could vary considerably and this could affect the sensitivity of the PCR. Including an internal control protocol for a constitutive gene in PCR is imperative in these types of studies. We used the amylin gene as a reporter for quality control in the present study.

Moreover, abnormal cell number is important, because it was previously observed that when <50 abnormal cells were found in the tissue, the possibilities of a false-negative outcome increased 26 times, compared with a higher cell count (200+ cells) [30].

Limitations

There are some limitations to consider when analyzing the results of this study. The prevalence of infection in our sample was too low to draw conclusions between the relationship between HPV and thyroid cancer. Additionally, the techniques used in the extraction and processing of DNA samples were chosen based on our available resources, and are not regarded as the best currently defined techniques. Future studies should increase the sample size as well as use more advanced DNA processing techniques in order to further define this relationship. Although these results need to be validated by future studies, we hope they work as an antecedent in identifying the relationship between HPV and thyroid neoplasms.

## Conclusions

HPV is a low-frequency finding in benign and malignant thyroid tumors. To date, there is no evidence to the best of our knowledge that confirms the relationship between HPV and thyroid tumors. Based on the high prevalence of this virus and its known role in cancer processes, it is necessary to search for a relationship between this virus and different common tumors in the population.

Due to the low prevalence of this virus in our sample, it is not possible to reach conclusions, however, the study and its bibliographic review we hope will serve as a reference to continue this research to confirm or reject this connection. The possibility of finding a relationship between this virus and an oncogenic pattern in the thyroid allows us to learn more about its oncongenic processes, as well as to develop detection tests and future treatment alternatives. The present study is one of the few that attempts to establish a link, but more evidence is needed to confirm or deny the link.
